# Temporin-Like Peptides Show Antimicrobial and Anti-Biofilm Activities against *Streptococcus mutans* with Reduced Hemolysis

**DOI:** 10.3390/molecules25235724

**Published:** 2020-12-04

**Authors:** Hanqi Wei, Zhipeng Xie, Xiuchuan Tan, Ran Guo, Yanting Song, Xi Xie, Rong Wang, Lushuang Li, Manchuriga Wang, Yingxia Zhang

**Affiliations:** 1 Key Laboratory of Tropical Biological Resources of Ministry of Education, School of Life and Pharmaceutical Sciences, Hainan University, Haikou 570228, China; w370694686@163.com (H.W.); xiezhipeng1994@163.com (Z.X.); xiuchuantan2020@163.com (X.T.); twtmqj9609@163.com (R.G.); songyanting3323@hotmail.com (Y.S.); xiexi@hainanu.edu.cn (X.X.); wang832820@126.com (R.W.); lvylushuang@163.com (L.L.); 2 College of Animal Science and Technology, Hainan University, Haikou 570228, China

**Keywords:** antimicrobial peptides, *Streptococcus mutans*, anti-biofilm activity

## Abstract

In our previous study, temporin-GHaR (GHaR) showed potent antimicrobial activity with strong hemolytic toxicity. To overcome its weakness, we designed GHaR6R, GHaR7R, GHaR8R, GHaR9R, and GHaR9W by changing the number of positive charges and the hydrophobic surface of GHaR. With the exception of GHaR7R, the hemolytic toxicity of the derived peptides had been reduced, and the antimicrobial activities remained close to the parent peptide (except for GHaR9R). GHaR6R, GHaR7R, GHaR8R, and GHaR9W exhibited a great bactericidal effect on *Streptococcus mutans* (*S. mutans*), which is one of the main pathogens causing dental caries. According to the membrane permeation and scanning electron microscope (SEM) analysis, these derived peptides targeted to the cell membranes of planktonic bacteria, contributing to the disruption of the membrane integrity and leakage of the intracellular contents. Moreover, they inhibited the formation of biofilms and eradicated the mature biofilms of *S. mutans*. Compared with GHaR7R, the derived peptides showed less cytotoxicity to human oral epithelial cells (HOECs). The derived peptides are expected to be the molecular templates for designing antibacterial agents to prevent dental caries.

## 1. Introduction

Dental caries is considered to be the most common oral disease and related to a variety of bacteria, of which *Streptococcus mutans* (*S. mutans*) is a significant contributor to tooth decay [1,2]. *S. mutans* produces many extracellular polysaccharides (EPS), which promotes the adhesion of bacteria on the surface of teeth and the co-aggregation with other microorganisms. EPS can also combine with various types of biopolymers derived from another bacterial metabolism to develop the multispecies biofilms [3]. Biofilms are highly organized microbial communities, in which the extracellular matrix provides a physical barrier for the bacteria and protect them from the antibiotics and environmental stress factors [4,5]. Meanwhile, when sugary and starchy foods are processed in the oral cavity, *S. mutans* converts carbohydrates into organic acids (acidogenicity), causing enamel damage [6]. At present, there are two commonly used methods of eradicating oral biofilms, including mechanical removal and drug dissolution [7]. Although they have been successfully used, the disadvantages still need to be overcome. Mechanical methods to remove dental plaque, such as brushing and flossing, are difficult to completely eradicate the mature biofilms in the gaps between teeth. The antibacterial agents regularly used in the oral cavity, chlorhexidine and fluoride, may induce bacterial resistance, teeth discoloration, and oral allergies [8,9,10].

Antimicrobial peptides (AMPs) are naturally occurring peptides found in many living organisms, which play an important role in the innate immune system. AMPs are usually positively charged, and exhibit remarkable antimicrobial activity. Most of them target the cell membrane of pathogenic microorganisms, which is a complex component for the bacterial cells to modify. Thus, pathogen bacteria rarely develop drug resistance to AMPs [11]. This characteristic makes AMPs the therapeutic drug candidates for infectious diseases. Recently, studies showed that AMPs exerted antimicrobial activities against *S. mutans*, such as MUC7 [12], ZXR-2 [13], and Bac8c [14].

In our previous study, we obtained temporin-GHa (GHa) from *Hylarana guentheri* skin, which was positively charged by histidine, and lacked antibacterial effect [15]. To investigate how the different positively charged amino acids impact the antibacterial activity, we designed and synthesized GHa analog, GHaR, in which the histidines were substituted with arginines. Although its antimicrobial activity had been greatly improved, the hemolysis was simultaneously enhanced. Considering that the antimicrobial activity and hemolytic toxicity of AMPs are related to the number of charges and hydrophobic surface [16], we designed GHaR6R, GHaR7R, GHaR8R, GHaR9R, and GHaR9W by replacing one amino acid residue in GHaR. Except for GHaR9R, these analogs showed the antimicrobial and anti-biofilm activities, and the hemolysis of GHaR6R, GHaR8R, and GHaR9W has been effectively reduced. In addition, we also performed membrane permeation and SEM analysis to explore the mechanism of the peptides against *S. mutans*. The effects of the peptides on biofilms were evaluated by crystal violet (CV) and SYTO staining. And the cytotoxicity of AMPs to HOECs was measured.

## 2. Results

### 2.1. Peptide Design and Structure Prediction

**Figure 1:**
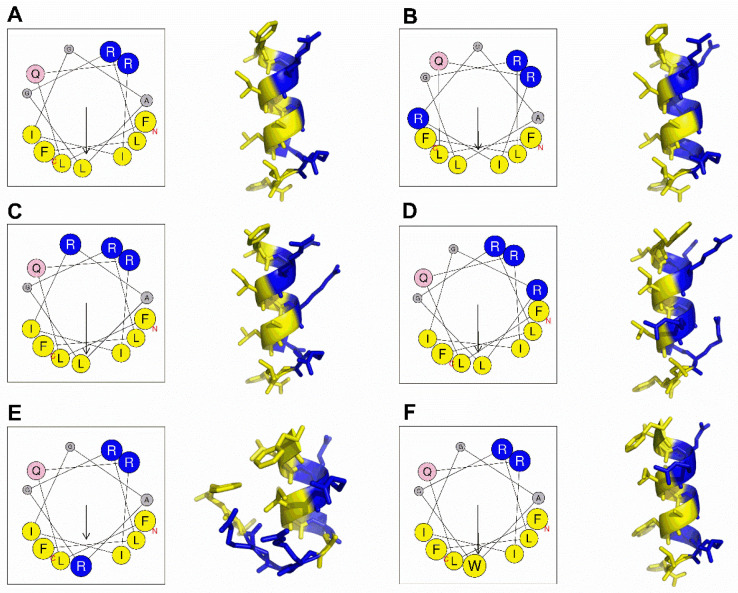
The structure prediction of (**A**) GHaR, (**B**) GHaR6R, (**C**) GHaR7R, (**D**) GHaR8R, (**E**) GHaR9R, and (**F**) GHaR9W. The helical wheels of peptides were predicted by Heliquest (https://heliquest.ipmc.cnrs.fr/). The arrows indicated the direction of the hydrophobic moment. Blue represented hydrophilic amino acid residues, yellow represented hydrophobic amino acid residues. The predicted 3D structures of GHaR and the derived peptides were analyzed by PEP-FOLD (https://bioserv.rpbs.univ-paris-diderot.fr/services/PEP-FOLD3/).

According to previous studies, the increase of an appropriate number of positively charged amino acid residues on AMPs can enhance their antibacterial activity, and effectively reducing the hydrophobicity decrease the hemolytic toxicity of AMPs [17,18]. Therefore, we chose arginine occurring in natural AMPs with high-frequency to replace one of the amino acids either on hydrophobic surface or hydrophilic surface to obtain GHaR6R, GHaR7R, GHaR8R, and GHaR9R. GHaR9W was obtained by displacing leucine (occurring on the hydrophobic surface in hemolytic peptides frequently) with tryptophan, which is the important residue of AMPs contributing to the destructive effect on the bacterial membrane [19]. As shown in Figure 1, the isoleucine and alanine at positions 6 or 8 of GHaR were replaced with arginine to obtain GHaR6R (Figure 1B) and GHaR8R (Figure 1D), reducing the hydrophobic surface of the peptides. In GHaR7R (Figure 1C), the glycine at position 7 was substituted with arginine to increase the positive charges on the hydrophilic surface. In GHaR9R (Figure 1E), the leucine was directly displaced at position 9 in the middle of the hydrophobic surface by arginine to destroy the hydrophobic surface of the peptide. In addition, GHaR9W (Figure 1F) was obtained by changing leucine to tryptophan. The structure prediction showed that the α-helical structure of GHaR9R was destroyed, while the other peptides maintained the α-helical structure.

### 2.2. Physicochemical Properties Analysis and Antimicrobial Activity Prediction

The sequences and the physicochemical properties of the peptides were shown in Table 1. The hydrophobic moment (μH) of GHaR9R was 0.554, which was the smallest among the derived peptides. Generally, the α-helical AMPs were expected to have positive grand average hydropathy (GRAVY) and negative or close to 0 Boman index (BI) [20]. The BI of the derived peptides was increased, and the GRAVY was decreased. As shown in Table S1, the probability scores of GHaR6R and GHaR9R predicted by the support vector machine (SVM), random forest (RF), and discriminant analysis (DA) models decreased, indicating that their antimicrobial activities were weaker than GHaR. The similar probability scores of the other peptides demonstrated that the antimicrobial activities were close to GHaR.

### 2.3. Hemolysis Assay

**Figure 2:**
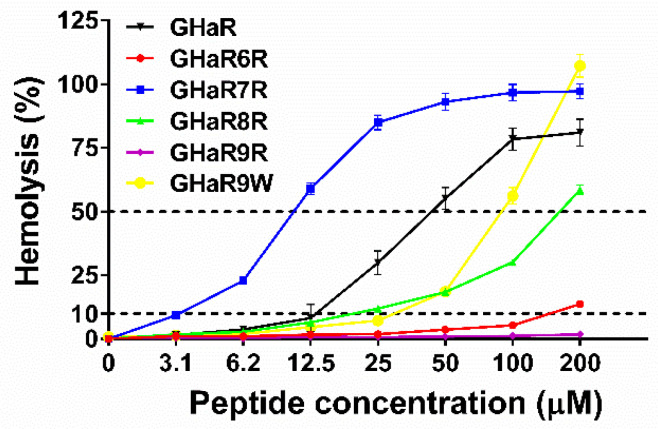
The hemolytic toxicity of GHaR and the derived peptides. The dashed line represented 10% and 50% hemolysis.

The hemolysis results of GHaR and the derived peptides were shown in Figure 2. The 50% hemolysis (HL_50_) of GHaR6R, GHaR8R, and GHaR9W were 1–5 times higher than that of GHaR, indicating that their hemolytic toxicities were significantly reduced. GHaR9R didn’t show hemolytic toxicity even when the concentration was raised up to 200 μM. However, GHaR7R exhibited much stronger hemolytic potency than the parent peptide. The cell selectivity index (CSI) of GHaR6R and GHaR8R were improved (Table S2). Especially, the CSI of GHaR8R reached the value of 27, which was about two times higher than that of the parent peptide.

### 2.4. Bacterial Susceptibility Test

Table 2 shows the minimum inhibitory concentrations (MICs) and the minimum bactericidal concentrations (MBCs) of GHaR and the derived peptides. Except for GHaR9R losing its antimicrobial activity, the derived peptides maintained the antibacterial activity of the parent peptide against Gram-positive bacteria, and the antibacterial effects of GHaR6R, GHaR7R, and GHaR8R on Gram-negative bacteria were even enhanced. Therefore, we selected GHaR6R, GHaR7R, GHaR8R, and GHaR9W for further studies to determine their activities against *S. mutans*.

### 2.5. Growth Inhibition Kinetics

**Figure 3:**
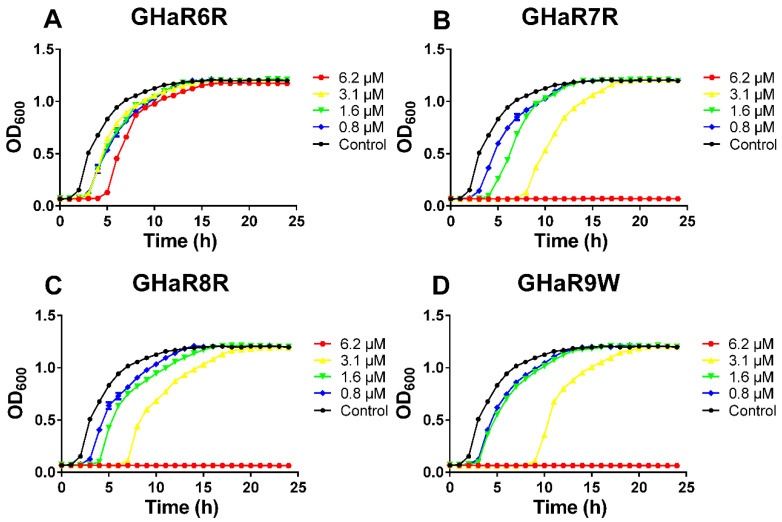
The growth inhibition curves of (**A**) GHaR6R, (**B**) GHaR7R, (**C**) GHaR8R, and (**D**) GHaR9W against *S. mutans* that were grown in Brain Heart Infusion Broth (BHI) for 24 h. Non-treated bacteria were served as the negative control.

We measured the growth inhibition curves of *S. mutans* within 24 h to assess whether the peptides had any effects on the growth of *S. mutans*. As shown in Figure 3, the lag phase of bacteria in the GHaR6R treatment group was prolonged by 3 h at a concentration of 6.2 μM, and there was no significant difference in bacterial growth between the treatment group and the untreated group up to 15 h. The growth of bacteria was completely inhibited by GHaR7R, GHaR8R, and GHaR9W at a concentration of 6.2 μM. The lag phase was extended by 6–8 h, and the stationary phase was delayed by 5–7 h after the bacteria were treated by those three peptides at 3.1 μM. However, when the peptides and bacteria were incubated for 20 h, the absorbance of peptide treatments at all concentrations (except for 6.2 μM) was similar to that of the negative control, indicating that the bacteria recovered.

### 2.6. Killing Kinetics

**Figure 4:**
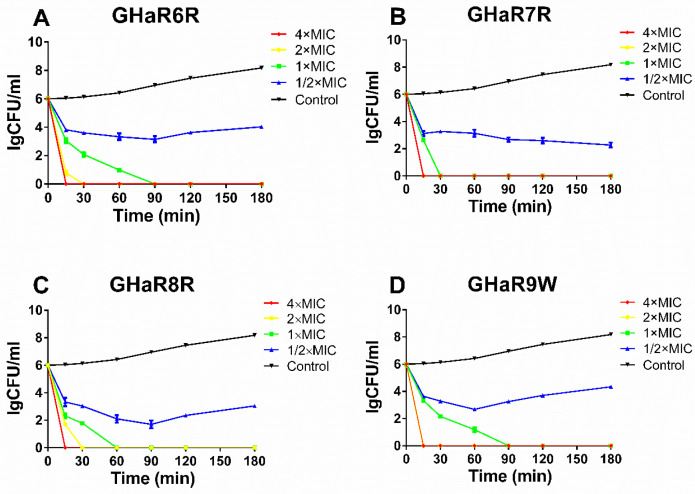
The killing assays of (**A**) GHaR6R, (**B**) GHaR7R, (**C**) GHaR8R, and (**D**) GHaR9W against *S. mutans* at 4 × MIC, 2 × MIC, 1 × MIC, and 1/2 × MIC. The bacteria untreated with the peptides served as the negative control.

The killing kinetics showed the antimicrobial activity and bactericidal efficiency of the peptides against bacteria. The time-killing curves of GHaR6R, GHaR7R, GHaR8R, and GHaR9W against *S. mutans* were shown in Figure 4. All peptides exerted antibacterial activity immediately after being added to the bacteria in a concentration-dependent and time-dependent manner. At a concentration of 4 × MIC, the four peptides completely eradicated the bacteria within 15 min. GHaR7R and GHaR9W showed stronger activities, and killed all the bacteria within 15 min at 2 × MIC. GHaR6R and GHaR8R killed the bacteria within 30 min at 2 × MIC.

### 2.7. Membrane Permeation Assay

**Figure 5:**
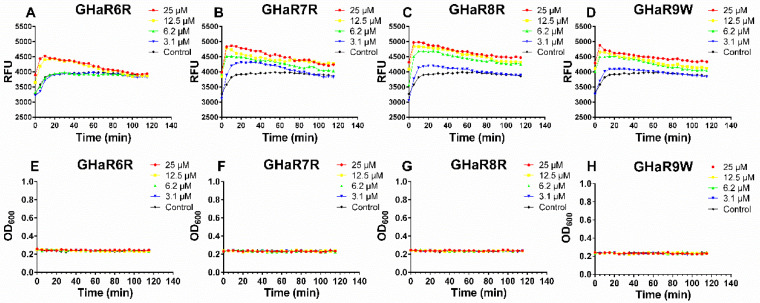
Membrane permeation after treatment with (**A**) GHaR6R, (**B**) GHaR7R, (**C**) GHaR8R, and (**D**) GHaR9W against *S. mutans* at 3.1–25 μM in the presence of propidium iodide. Relative fluorescence units (*y*-axis) were abbreviated as RFU in the figures. The growth inhibition curves of (**E**) GHaR6R, (**F**) GHaR7R, (**G**) GHaR8R, and (**H**) GHaR9W against *S. mutans* at 3.1–25 μM within 2 h. The bacteria untreated with the peptides served as the negative control.

Propidium iodide is known to enter the bacteria only when the cell membranes have been destroyed, and the fluorescence is detected after binding with DNA. The membrane permeation effect of the peptides was determined by measuring the fluorescence intensity [21]. As shown in Figure 5A–D, the fluorescence intensity reached maximum value within 5–10 min. The intake of propidium iodide induced by GHaR6R at a concentration of 3.1 μM and 6.2 μM were similar to the group in the absence of the peptide. For GHaR7R, GHaR8R, and GHaR9W, with a concentration of 3.1 μM, the fluorescence intensity increased slightly. When the bacteria were treated with the peptides at higher concentrations, the fluorescence intensity increased significantly. The growth inhibition curves of the bacteria showed that *S. mutans* did not proliferate within 2 h after treatment with the peptides at a concentration of 3.1 to 25 μM (Figure 5E–H).

### 2.8. Scanning Electron Microscopy Analysis

**Figure 6:**
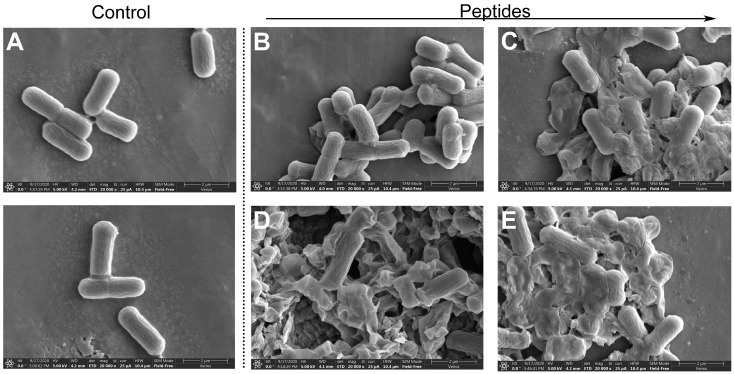
Morphological changes of *S. mutans* exposed to (**B**) GHaR6R, (**C**) GHaR7R, (**D**) GHaR8R, and (**E**) GHaR9W with a concentration of 25 μM. The bacteria were treated with PBS served as (**A**) the negative control. SEM magnification, × 20,000. The scale was 2 μm.

SEM was used to observe the morphological changes of *S. mutans* treated by GHaR6R, GHaR7R, GHaR8R, and GHaR9W for 1 h. As shown in Figure 6, *S. mutans* in the untreated group had smooth surfaces and a rod-like shape. After exposed to GHaR6R, the surfaces of the bacteria were rough and obviously wrinkled. Most of the bacteria treated by GHaR7R, GHaR8R, and GHaR9W were severely damaged and lost the normal bacterial morphology. The cell membranes were disrupted completely and attached to each other, causing the leakage of the intracellular contents.

### 2.9. Anti-Biofilm Activity

**Figure 7:**
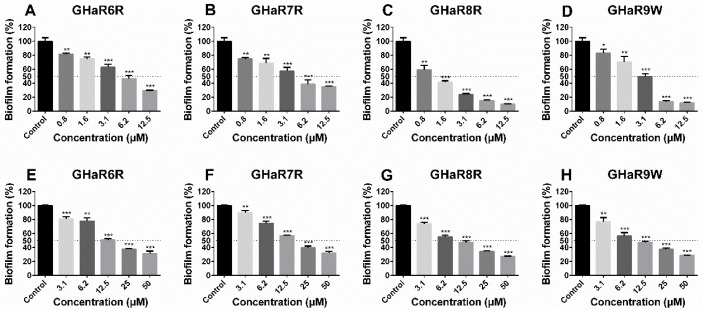
The biofilms formation inhibitory activity of (**A**) GHaR6R, (**B**) GHaR7R, (**C**) GHaR8R, and (**D**) GHaR9W, and the mature biofilms eradication of (**E**) GHaR6R, (**F**) GHaR7R, (**G**) GHaR8R, and (**H**) GHaR9W. The biofilms were stained by CV, and the absorbance was monitored at 590 nm. (* *p* < 0.05, ** *p* < 0.01, *** *p* < 0.001). The biofilms untreated with the peptides were used as the negative control. The dashed lines represented 50% of the biofilm formation.

The derived peptides effectively inhibited the formation of *S. mutans* biofilms in a concentration-dependent pattern (Figure 7A–D). At a concentration of 12.5 μM, the inhibition rates of GHaR8R and GHaR9W reached up to 90.0% and 88.2%, and the MBIC_50_ of them were 1.6 μM and 3.1 μM (Table S3). GHaR6R and GHaR7R showed a less inhibitory effect on the biofilms formation at the same concentration with the inhibition rates of 70.5% and 65.2%, and the MBIC_50_ was 6.2 μM. However, all peptides exhibited similar eradicated ability on mature biofilms (Figure 7E–H). When the concentration was 50 μM, the eradication rate was 68.2%–73.1%. The MBEC_50_ of GHaR8R and GHaR9W were 12.5 μM.

### 2.10. Fluorescence Microscope Analysis

**Figure 8:**
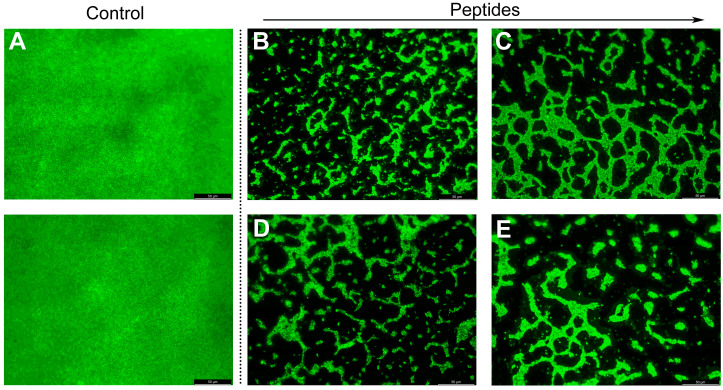
Fluorescence microscope image of mature biofilms of *S. mutans* stained with SYTO (green) after treatment with (**B**) GHaR6R, (**C**) GHaR7R, (**D**) GHaR8R, and (**E**) GHaR9W at 50 μM. The biofilms untreated with the peptides were used as (**A**) the negative control.

The mature biofilms treated with the derived peptides were stained with SYTO and observed by the fluorescence microscope (Figure 8). The biofilms without peptides treatment were dense and thick. The biofilms can be effectively destroyed and eradicated by the peptides at 50 μM. Especially after exposed to GHaR8R and GHaR9W, only the dispersed clusters of the biofilms were observed.

### 2.11. Viability of Human Oral Epithelial Cells

**Figure 9:**
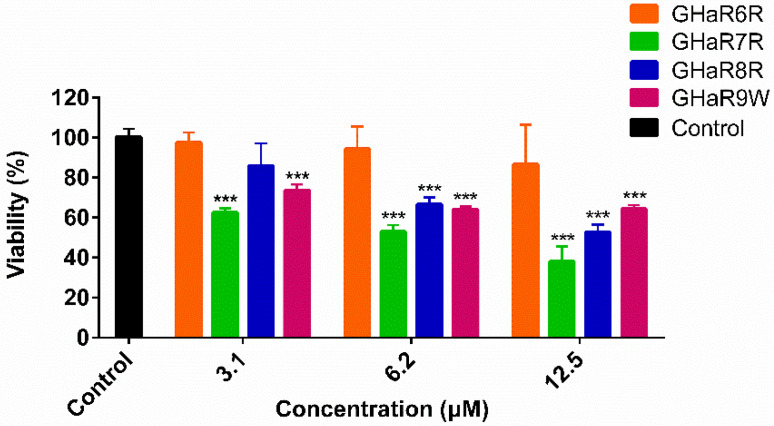
Cytotoxicity of GHaR6R, GHaR7R, GHaR8R, and GHaR9W. The viability of HOECs treated with the peptides at 3.1–12.5 μM was determined by using CCK-8. The negative control was treated with PBS only. (* *p* < 0.05, ** *p* < 0.01, *** *p* < 0.001).

As shown in Figure 9, compared with the negative control, GHaR6R had no obvious effect on the viability of HOECs at the highest concentration of 12.5 μM. The half-maximal inhibitory concentration (IC_50_) of GHaR6R was 39.0 μM. Comparatively, GHaR7R showed cytotoxicity at the lowest concentration of 3.1 μM. GHaR8R inhibited cell growth slightly at low concentration. When the concentration was up to 6.2 μM, GHaR8R and GHaR9W exhibited similar cytotoxicity on the cells, with the viability of 66.7% and 64.1%.

## 3. Discussion

At present, AMPs are becoming a resource of promising antimicrobial agents, and more and more methods, such as library screening, template-based design, database-assisted design, and structure-based design, have been used in the artificial development of AMPs [22]. Our previous studies mentioned that the derived peptides (GHaK, GHa4K, GHa11K) obtained by replacing histidine with lysine on GHa showed better antibacterial activities than the mother peptide [23]. Meanwhile, GHaR was obtained by replacing histidine with arginine. GHaR exhibited remarkable antibacterial activities, but the hemolysis was strong. Therefore, we chose GHaR as the template combining with database-assisted design to optimize temporin peptides with great antimicrobial activity and lower cytotoxicity by changing the number of positive charges or hydrophobic surface.

Studies had shown that the physicochemical properties, including charge number, hydrophobicity, μH, and isoelectric point (PI), impact the activity of α-helical AMPs. It was reported that increasing the positive charges within 10 may enhance the antimicrobial activity of AMPs, while the hemolytic toxicity was insignificantly affected [16,24]. However, optimizing the antibacterial effect of AMPs by increasing the number of charges is limited. The number of positive charges of the GHaR6R, GHaR7R, and GHaR8R had increased from 2 to 3. Their antibacterial activity is not stronger than GHaR. Especially for GHaR7R, the hydrophobic surface is similar to GHaR, and the significant difference between it and the parent peptide is the number of positive charges. Therefore, it is deduced that the increase in positive charges might reduce the self-aggregation ability of AMPs. This is consistent with the results of previous studies. When the positive charge number of L-V13K was modified from 9 to 10, the increased electrostatic repulsion between the AMPs is greater than the electrostatic attraction of the AMPs to the cell membranes, which reduces the antimicrobial activity [25]. Dathe et al. demonstrated that increasing the charge number of magainin 2 from 4 to 5 enhanced the antimicrobial activity to the maximum. If the charges increased to 7, the activity was not improved anymore [26]. Therefore, there is no obvious general correlation between antibacterial activity and the number of charges.

The hemolytic tendency was different among the derived peptides. The enhanced hemolytic toxicity of GHaR7R may be concerned with the increase in μH. The hemolysis of GHaR6R, GHaR8R, and GHaR9W had significantly been reduced. Especially for GHaR8R, the CSI value was 27. In addition, previous studies had also shown that hemolytic toxicity can be decreased by reducing the hydrophobic surface or introducing charged residues to destroy the hydrophobic surface [27]. The hydrophobic surface of GHaR9R was disturbed by directly replacing the leucine on the hydrophobic surface with an arginine. Although it has no hemolytic toxicity, its antimicrobial activity was lost, which meant that the α-helix with a hydrophobic surface was the most important factor in the antimicrobial activity. This provides us with an inspiration that it is difficult to completely overcome cytotoxicity, while improving the antimicrobial activity of AMPs, and a balance point might be achieved to minimize the cytotoxicity to the greatest extent possible.

The MICs of GHaR7R, GHaR8R, and GHaR9W were the same, but their bactericidal efficiencies were different. GHaR7R had the highest bactericidal efficiency. Their bactericidal properties can effectively prevent the regeneration of *S. mutans*. Most AMPs acted on cell membranes rather than specific receptors of bacteria [28]. Our results demonstrated that the derived peptides targeted the bacterial membranes, leading to the damage of membranes and significant changes in the morphology of *S. mutans*. We deduced that the positively charged AMPs interacted with the negatively charged cell membranes to destroy the membrane integrity and exert their bactericidal effects on the planktonic bacteria. Dental caries is considered to be a disease caused by the accumulation of bacterial biofilms on teeth surface [29]. The derived peptides effectively inhibited the formation of biofilms and eradicated mature biofilms of *S. mutans*. Combined with the analysis of bactericidal kinetics, the peptides at a concentration of 1 × MIC have limited toxicity to HOECs.

In summary, GHaR6R, GHaR7R, GHaR8R, and GHaR9W not only exerted antimicrobial activity by destroying cell membranes of planktonic bacteria, but also exhibited anti-biofilm activity. Because of the strong hemolysis and cytotoxicity to HOECs of GHaR7R, the low-toxicity peptides of GHaR6R, GHaR8R, and GHaR9W are expected to become promising antimicrobial candidates to develop anti-caries agents. Our research also provides a theoretical basis for the design of high-efficiency and low-toxicity AMPs.

## 4. Materials and Methods

### 4.1. Materials

The strains were used in the experiments, including Gram-positive bacteria *Staphylococcus aureus* (ATCC 25923), *Bacillus subtilis* (ATCC 6633), *Streptococcus mutans* (ATCC 25175), Methicillin-resistant *Staphylococcus aureus* (ATCC 43300), Methicillin-resistant *Staphylococcus aureus* 1–3 (clinically isolated, number 1–3), Gram-negative bacteria *Escherichia coli* (ATCC 25922), *E. coli* (D 31), *Pseudomonas aeruginosa* (ATCC 15442), *P. aeruginosa* PAO1 (wild type), and the fungus *Candida albicans* (ATCC 10231). *S. mutans* was cultured in Brain Heart Infusion Broth (BHI, Beijing Land Bridge, China) under anaerobic condition; *C. albicans* was grown in Sabouraud Dextrose Broth (SDB, HuanKai Microbial, Guangzhou, China); and the other bacterial strains were grown in Tryptic Soy Broth (TSB, HuanKai Microbial, Guangzhou, China). All bacteria and fungi were cultivated to the logarithmic phase at 37 °C before experiments were performed.

### 4.2. Peptide Design and Structure Prediction

Heliquest (https://heliquest.ipmc.cnrs.fr/) predicts the helix diagram of the peptides. The PEP-FOLD (https://bioserv.rpbs.univ-paris-diderot.fr/services/PEP-FOLD3/) predicts the 3D model, and the visualization software PyMol (1.5.0.3) displays the 3D model [23].

### 4.3. Physicochemical Properties and Antimicrobial Activity Analysis

The analysis tools of Heliquest (https://heliquest.ipmc.cnrs.fr/), ExPasy (https://web.expasy.org/compute_pi/) and the database of APD3 (http://aps.unmc.edu/AP/main.php) were used to analyze μH, charge number, PI, BI, and GRAVY of the AMPs to assist in the design of peptides [23]. CAMP_R3_ (http://www.camp.bicnirrh.res.in/prediction.php) is an AMP prediction tool, which combines amino acid sequence, physicochemical properties, and structural features. AMPs are analyzed through four different models, SVM, RF, artificial neural network (ANN), and DA. SVM, RF, and DA models give probability scores (0–1) of antibacterial potencies. The larger the value, the more likely the peptide exerts antimicrobial activity. The predicted results of the ANN model show that AMP is an antibacterial sequence, and NAMP refers to a sequence that does not produce antibacterial effects.

### 4.4. Synthesis and Storage of Peptides

GHaR and the derived peptides were synthesized by Jier Biochemical Co., Ltd. (Shanghai, China) using solid-phase synthesis. The peptides were purified by reverse-phase high-performance liquid chromatography (RP-HPLC, NP7000 C, Hanbon Sci&Tech, Jiangsu, China) with a purity greater than 95% (Figures S1–S6) and stored at −80 °C before use.

### 4.5. Hemolysis Assay

The hemolytic toxicity of the peptides was determined as described [30]. Human red blood cells (hRBCs) were washed with phosphate-buffered saline (PBS, pH 7.2) and centrifuged at 1000× *g* for 10 min at 4 °C until the supernatant was clear. 4% hRBCs and an equal volume of the peptides with different concentrations were incubated at 37 °C for 1 h. After incubation, the supernatant was collected by centrifugation, and 150 μL was transferred to a new 96-well plate (Corning, New York, NY, USA). The absorbance at 450 nm was measured. The 0.1% Triton X-100 was used as the positive control, and PBS was used as the negative control. The minimum hemolysis concentration (MHC) was defined as the lowest concentration of peptide that causes 10% hemolysis, and HL_50_ was the lowest concentration that causes 50% hemolysis. CSI was defined as the ratio of HL_50_ to MIC [31].

### 4.6. Antimicrobial Activity Detection

The antimicrobial activities of the peptides were measured by the two-fold dilution method [32]. Each strain was inoculated in the medium and cultivated to the logarithmic phase. A series of diluted peptides (concentrations of 3.1–100 μM, 50 μL) were prepared in a 96-well plate (Corning, New York, NY, USA), and the same volume of the bacterial suspension (the final concentration of 1 × 10^6^ CFU/mL) was added. After incubation at 37 °C for 18–24 h, the absorbance at 600 nm was measured by using a microplate reader (Multiskan Spectrum, BioTek, Winuski, VT, USA). MIC was the lowest concentration of peptides that completely inhibited bacterial growth. All tests were performed in triplicate independent experiments.

From the MIC measuring plates, 50 μL of bacteria suspension in the presence of peptides when the concentrations were equal and up to MICs was spread on the BHI agar medium, and incubated at 37 °C for 24 h. The peptide concentration with no bacterial growth was defined as MBC [33].

### 4.7. Influence on Growth of S. mutans

The growth inhibition curve demonstrated the influence of the peptides on bacterial growth by measuring the changes in absorbance at 600 nm within 24 h [34]. An equal volume of bacterial solution (the final concentration of 1 × 10^6^ CFU/mL) was added to the prepared peptides (the concentrations of 1.6–12.5 μM) in a 96-well plate. Non-treated bacteria were served as the negative control. The total volume of the mixture was 200 μL. The plate was incubated in a microplate reader (Multiskan Spectrum, BioTek, Winuski, VT, USA) at 37 °C, and the absorbance was measured every hour for 24 h.

### 4.8. Time-Killing Curves

Plate coating was used to determine the time-killing curves [35,36]. The bacteria were cultivated to the logarithmic phase, and the peptides were diluted to the concentrations of 1×, 2×, 4×, and 8× MIC. The same volume of the bacterial suspension (the final concentration of 1 × 10^6^ CFU/mL) and the peptides were mixed, and incubated at 37 °C under anaerobic conditions for 0, 15, 30, 60, 90, 120, and 180 min respectively. After incubation, the mixture was diluted, and 50 μL of the bacterial suspension was coated on the BHI agar. The bacterial colonies were counted after incubation at 37 °C for 12 h.

### 4.9. Propidium Iodide Uptake Assay

As previously described [22,37], *S. mutans* was cultured to the logarithmic phase. The bacteria were harvested and washed three times with PBS (pH 7.2). The bacteria (the final concentration of 1 × 10^8^ CFU/mL) were seeded in a 96-well plate (Corning, New York, NY, USA). And the peptides (the final concentrations of 3.1–25 μM) were added to each well, as well as propidium iodide (the final concentration of 20 μM). The mixtures were mixed thoroughly, and the fluorescence was monitored at an excitation wavelength of 584 nm and an emission wavelength of 620 nm every 5 min for 2 h by using the microplate reader (Spark, Tecan, Männedorf, Switzerland). Meanwhile, the bacterial growth was monitored at 600 nm. The graphs were drawn with an average of three independent experiments.

### 4.10. Scanning Electron Microscopy

The experiment was carried out with a slight modification according to the previous method [38]. After *S. mutans* (1 × 10^9^ CFU/mL) was incubated in the presence of the peptides (25 μM) at 37 °C for 1 h, the bacteria were fixed with 2.5% glutaraldehyde, followed by washing with PBS (pH 7.2) three times. After dehydration with ethanol at the concentrations of 30%, 50%, 70%, 90%, and 100%, the bacteria were resuspended in absolute ethanol, freeze-dried overnight, and then sprayed with gold. *S. mutans* was observed by SEM (Verios G4 UC, thermo scientific, Waltham, MA, USA). The bacteria were treated with PBS served as the negative control.

### 4.11. Effect of the Peptides on Biofilms

CV was used to detect the effects of the peptides on the formation of biofilm and the eradication of mature biofilm [39,40]. 100 μL of *S. mutans* in the logarithmic phase was diluted in BHI (containing 1% sucrose) to the concentration of 2 × 10^6^ CFU/mL. The suspensions were added to the equal volume of the peptides (the final concentrations of 0.8–12.5 μM). After incubation at 37 °C under anaerobic conditions for 24 h, the supernatant was discarded. The plate was irrigated with PBS carefully to wash away the planktonic bacteria. After fixing with anhydrous methanol for 15 min, 0.5% CV was added to each well for staining the biofilms. The excess CV was washed away by PBS (pH 7.2), and the bound CV was dissolved with anhydrous methanol. A microplate reader (Multiskan Spectrum, BioTek, Winuski, VT, USA) was used to measure the absorbance value of 590 nm. MBIC_50_ was defined as the lowest peptide concentration that inhibited 50% of biofilm formation [41].

To evaluate the ability of the peptides to eradicate the mature biofilms of *S. mutans*, the bacteria (the final concentration of 1 × 10^6^ CFU/mL) were seeded in a flat bottom 96-well plate for 24 h to obtain the mature biofilms. The planktonic bacteria were washed away, and the peptides with the concentrations of 3.1–50 μM were added. After treatment for 24 h, CV was used for staining and quantification as described above. The biofilms untreated with the peptides served as the negative control. MBEC_50_ was defined as the lowest peptide concentration that removed 50% of the mature biofilm [41].

### 4.12. Biofilm Observation by Fluorescence Microscope

To observe the eradicative effect of the peptides on the mature biofilms more intuitively, we observed the biofilms with a fluorescence microscope after SYTO staining [37]. The peptides with a final concentration of 50 μM were added to the mature biofilms. The biofilms untreated with the peptides served as the negative control. After incubation at 37 °C for 24 h, 10 μM SYTO was added to the stain for 15 min in the dark. The biofilms were observed with a fluorescence microscope (DM6000, Leica, Wetzlar, Germany), which equipped with a 40 × objective and a 10 × eyepiece.

### 4.13. Cytotoxicity Assay

The Cell Counting Kit-8 (CCK-8) (Beyotime Biotechnology, Shanghai, China) was used to determine the cytotoxicity [42]. The HOECs were obtained from Shrdio Company (Nanjing, China). The cells were resuscitated in Dulbecco’s modified Eagle’s medium (DMEM) containing 10% fetal bovine serum (FBS) and cultured in a 5% CO_2_ incubator at 37 °C. We diluted HOECs to a concentration of 1 × 10^5^ cells/mL, and 100 μL of cell suspension was added to each well of a 96-well plate. After incubated for 24 h, the cells were exposed to the peptides at different concentrations (3.1, 6.2, and 12.5 μM) for 90 min. The negative control was treated with PBS only. The peptides were removed, and fresh medium were added. After incubation for 24 h, 10 μL of CCK-8 was added to each well, and incubated for 4 h. The absorbance at 450 nm was measured. Three experiments were conducted independently. Before this experiment, the effects of DMEM on the activities of the peptides have been evaluated, and the results showed no effect.

### 4.14. Statistical Analysis

The Graphpad Prism 6 (GraphPad Software, Inc., La Jolla, CA, USA) was used for data analysis, and statistical significance was calculated with t test by comparison with the negative control (* *p* < 0.05; ** *p* < 0.01; *** *p* < 0.001). All experiments were conducted in triplicate.

## Figures and Tables

**Table 1:** Amino acid sequences and physicochemical properties of GHaR and the derived peptides.

Peptides	Sequence	MW ^a^	μH ^b^	Charge ^a^	PI ^c^	BI ^a^	GRAVY ^a^
GHaR	FLQRIIGALGRLF	1503.86	0.762	2	12.0	0.08	1.115
GHaR6R	FLQRIRGALGRLF	1546.89	0.693	3	12.3	1.61	0.423
GHaR7R	FLQRIIRALGRLF	1603.00	0.837	3	12.3	1.30	0.800
GHaR8R	FLQRIIGRLGRLF	1588.97	0.780	3	12.3	1.37	0.631
GHaR9R	FLQRIIGARGRLF	1545.89	0.554	3	12.3	1.61	0.477
GHaR9W	FLQRIIGAWGRLF	1575.91	0.804	2	12.0	0.28	0.754

^a^ Determined at APD3 (http://aps.unmc.edu/AP/main.php), MW is molecular weight, BI is the Boman index (kcal/mol), GRAVY is grand average hydropathy. ^b^ Determined by using Heliquest (https://heliquest.ipmc.cnrs.fr/), μH is the hydrophobic moment, ^c^ Determined by using ExPasy (https://web.expasy.org/compute_pi/), PI is the isoelectric point.

**Table 2:** MICs and MBCs of GHaR and the derived peptides on tested strains.

Species	Strains	MIC/MBC (μM)					
		GHaR	GHaR6R	GHaR7R	GHaR8R	GHaR9R	GHaR9W
Gram+	*S. aureus*	1.6/1.6	3.1/12.5	3.1/3.1	1.6–3.1/3.1	6.2/12.5	3.1/6.2
	*B. subtilis*	12.5/25	25/50	>50/>50	12.5/12.5	>50/>50	12.5/50
	*S. mutans*	3.1/6.2	12.5/12.5	6.2/12.5	6.2/6.2	>50/>50	6.2/6.2
	MRSA	3.1/3.1	6.2/12.5	3.1/6.2	3.1/3.1	>50/>50	6.2/12.5
	MRSA-1	3.1/25	12.5/25	6.2/25	6.2/6.2	>50/>50	6.2/12.5
	MRSA-2	6.2/12.5	12.5/25	6.2/50	3.1/12.5	>50/>50	6.2/12.5
	MRSA-3	3.1/6.2	6.2/25	6.2/25	6.2/12.5	>50/>50	6.2/12.5
Gram-	*E. coli*	6.2/6.2	12.5/12.5	3.1/12.5	3.1/3.1	>50/>50	12.5/12.5
	D31	12.5/25	25/50	12.5/50	12.5/25	>50/>50	12.5/50
	*P. aeruginosa*	6.2/6.2	>50/>50	>50/>50	50/>50	>50/>50	>50/>50
	PAO1	25/50	3.1/25	>50/>50	6.2/12.5	>50/>50	25/50
Fungi	*C. albicans*	12.5/50	50/50	25/25	12.5/12.5	>50/>50	25/25

*S. aureus*, *Staphylococcus aureus* (ATCC 25923); *B. subtilis*, *Bacillus subtilis* (ATCC 6633); *S. mutans*, *Streptococcus mutans* (ATCC 25175); MRSA, methicillin-resistant *S. aureus* (ATCC 43300); MRSA-1-3, methicillin-resistant *S. aureus* (clinically isolated, No.1-3); *E. coli*, *Escherichia coli* (ATCC 25922); D31, *E. coli* (D31) is an anti-streptomycin strain; *P. aeruginosa*, *Pseudomonas aeruginosa* (ATCC 15442); PAO1, *P. aeruginosa* PAO1 (wild type); *C. albicans*, *Candida albicans* (ATCC 10231).

## Supplementary Materials

The following are available online. Table S1: Antimicrobial activity prediction by CAMP_R3_, Table S2: Hemolytic toxicity analysis, Table S3: Anti-biofilm activity analysis, Figure S1: The mass spectrum (A) and RP-HPLC chromatogram (B) of GHaR, Figure S2: The mass spectrum (A) and RP-HPLC chromatogram (B) of GHaR6R, Figure S3: The mass spectrum (A) and RP-HPLC chromatogram (B) of GHaR7R, Figure S4: The mass spectrum (A) and RP-HPLC chromatogram (B) of GHaR8R, Figure S5: The mass spectrum (A) and RP-HPLC chromatogram (B) of GHaR9R, Figure S6: The mass spectrum (A) and RP-HPLC chromatogram (B) of GHaR9W.
