# Supplementary material for: Temporin-Like Peptides Show Antimicrobial and Anti-Biofilm Activities against Streptococcus mutans with Reduced Hemolysis
Source: Molecules. 2020 Dec 4;25(23):5724. doi: 10.3390/molecules25235724 (PMC7730238; doi:10.3390/molecules25235724)
Supplement: Supplementary file 1 [file molecules-25-05724-s001.pdf]

molecules

Temporin-like peptides show antimicrobial and anti-biofilm activities against *Streptococcus mutans* with reduced hemolysis

Hanqi Wei [1,†], Zhipeng Xie [1,†], Xiuchuan Tan [1], Ran Guo [1], Yanting Song [1], Xi Xie [1], Rong Wang[1], Lushuang Li [1], Manchuriga Wang [2,*] and Yingxia Zhang [1,*]

[1] Key Laboratory of Tropical Biological Resources of Ministry of Education, School of Life and Pharmaceutical Sciences, Hainan University, Haikou 570228, China; w370694686@163.com; xiezhipeng1994@163.com; xiuchuantan2020@163.com; twtmqj9609@163.com; songyanting3323@hotmail.com; xiexi@hainanu.edu.cn; wang832820@126.com; lvylushuang@163.com

[2] College of Animal Science and Technology, Hainan University, Haikou 570228, China

[*] Correspondence: manchuriga@hainanu.edu.cn; zhangyingxia@hainanu.edu.cn

[†] These authors contributed equally to this work

Received: date; Accepted: date; Published: date

TABLE OF CONTENTS:

Table S1: Antimicrobial activity prediction by $CAMP_{R3}$ 2
Table S2: Hemolytic toxicity analysis 2
Table S3: Anti-biofilm activity analysis 2
Figure S1. The mass spectrum (A) and RP-HPLC chromatogram (B) of GHaR 3
Figure S2: The mass spectrum (A) and RP-HPLC chromatogram (B) of GHaR6R 4
Figure S3. The mass spectrum (A) and RP-HPLC chromatogram (B) of GHaR7R 5
Figure S4: The mass spectrum (A) and RP-HPLC chromatogram (B) of GHaR8R 6
Figure S5: The mass spectrum (A) and RP-HPLC chromatogram (B) of GHaR9R 7
Figure S6: The mass spectrum (A) and RP-HPLC chromatogram (B) of GHaR9W 8

Table S1. Antimicrobial activity prediction by $CAMP_{R3}$

Peptides	SVM	RF	ANN	DA
GHaR	0.911	0.911	AMP	0.989
GHaR6R	0.877	0.5355	AMP	0.97
GHaR7R	0.979	0.89	AMP	0.995
GHaR8R	0.973	0.892	AMP	0.994
GHaR9R	0.88	0.778	AMP	0.981
GHaR9W	0.866	0.9385	AMP	0.917

Table S2. Hemolytic toxicity analysis

Peptides	MHC [a] (μM)	HL_{50} [b] (μM)	CSI [c]
GHaR	13.6	46.4	15
GHaR6R	156	>200	>16
GHaR7R	11.6	10.9	1.8
GHaR8R	22.3	167.6	27
GHaR9R	>200	>200	—
GHaR9W	32.8	86.3	13.9

[a] The minimum hemolytic concentration that caused 10% hemolysis of hRBCs; [b] The concentration at which 50% of hRBCs were lysed; [c] CSI was calculated by using the ratio of HL_{50} to the MIC of the peptides against *S. mutans*.

Table S3. Anti-biofilm activity analysis

Peptides	$MBIC_{50}$ (μM)	$MBEC_{50}$ (μM)
GHaR6R	6.2	25
GHaR7R	6.2	25
GHaR8R	1.6	12.5
GHaR9W	3.1	12.5

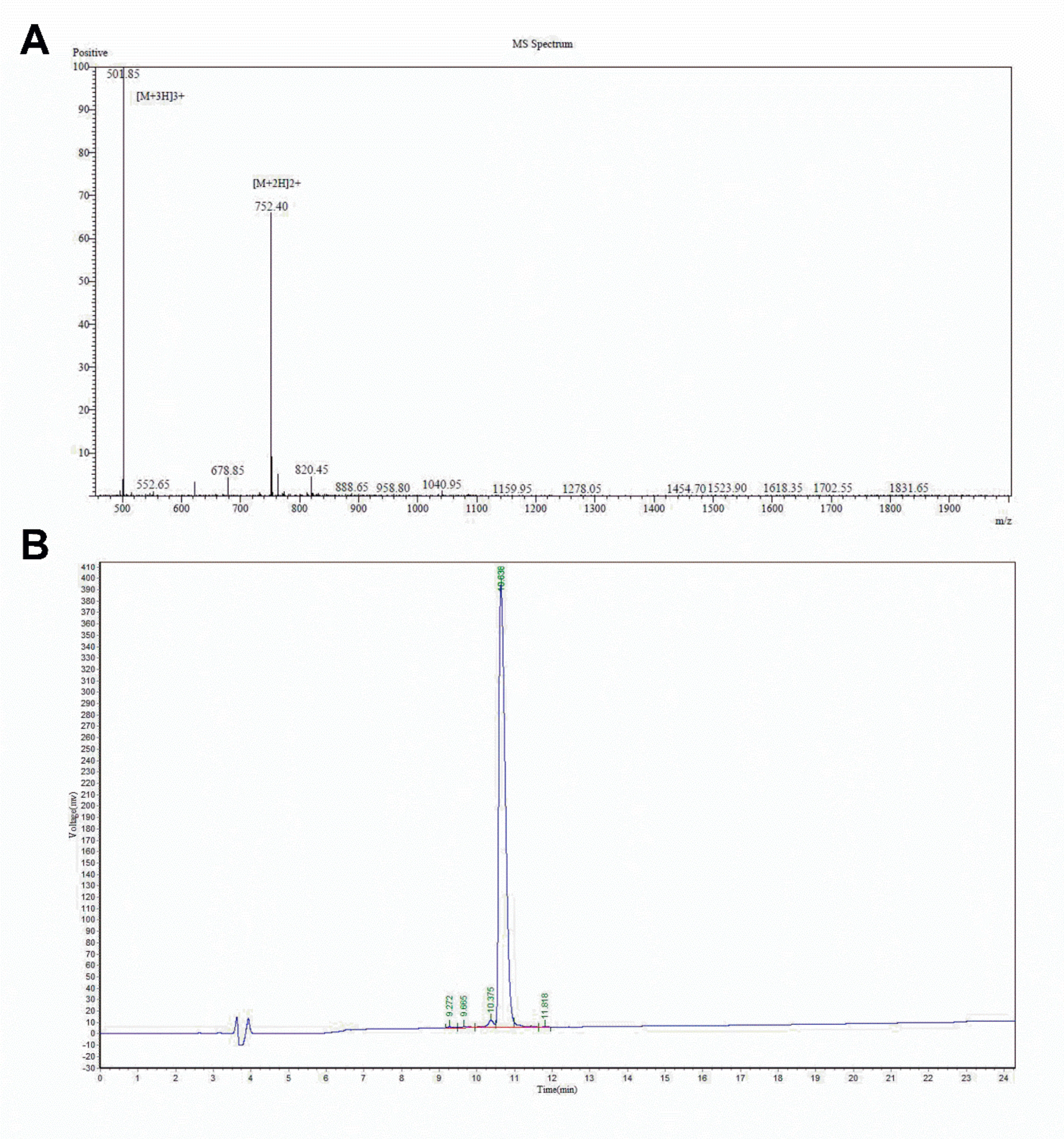

Figure S1. The mass spectrum (A) and RP-HPLC chromatogram (B) of GHaR.

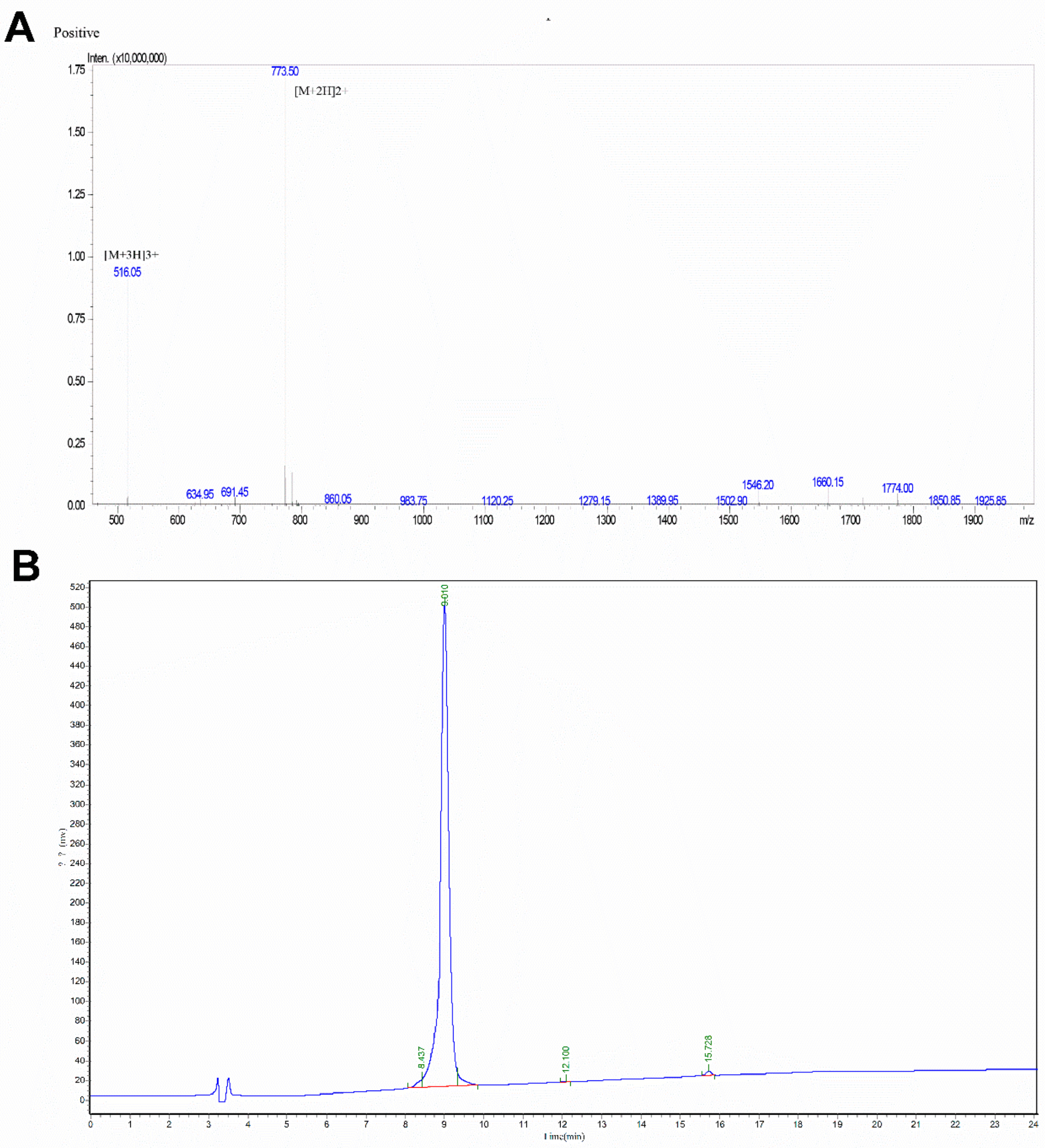

Figure S2. The mass spectrum (A) and RP-HPLC chromatogram (B) of GHaR6R.

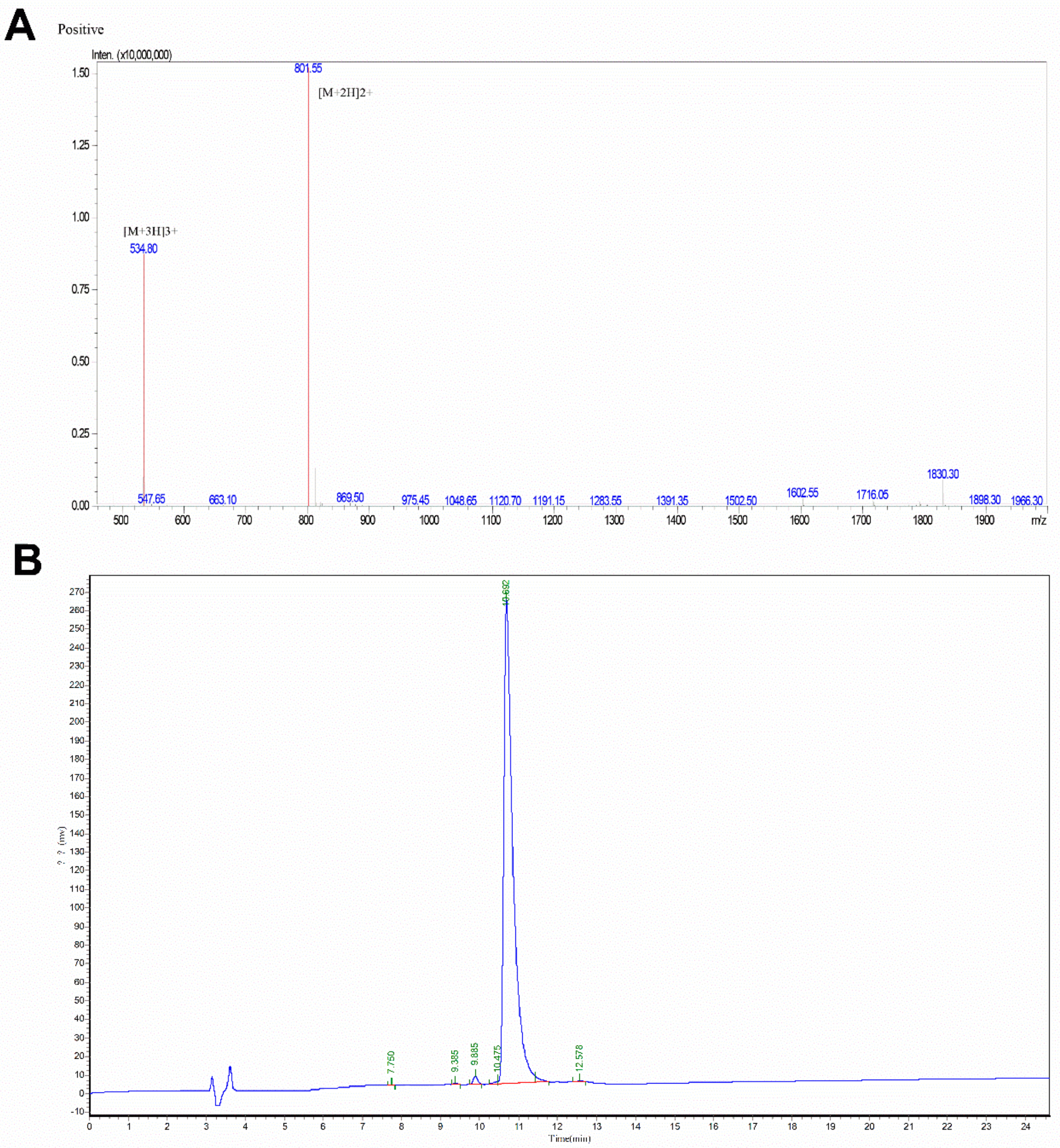

Figure S3. The mass spectrum (A) and RP-HPLC chromatogram (B) of GHaR7R.

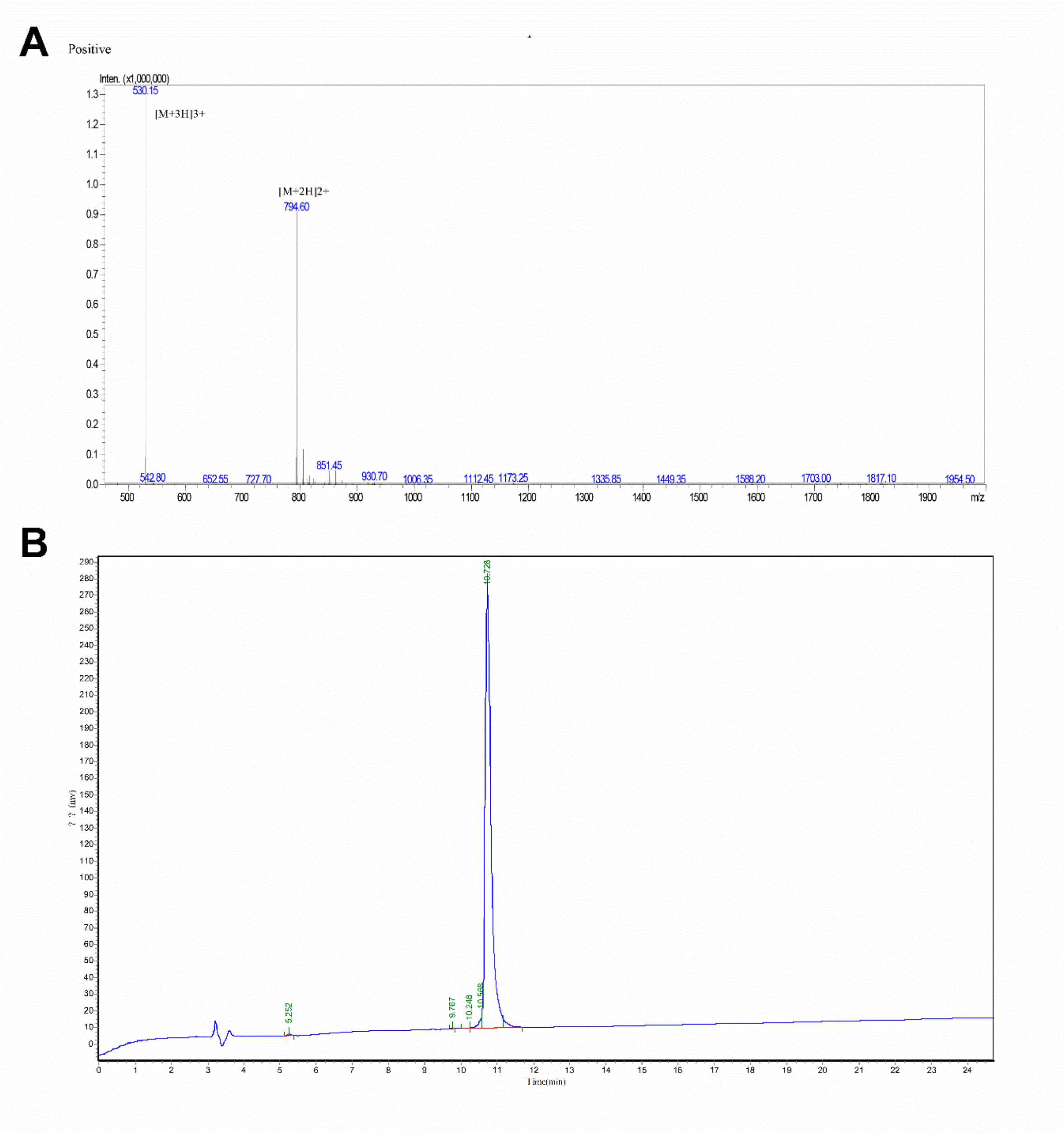

Figure S4. The mass spectrum (A) and RP-HPLC chromatogram (B) of GHaR8R.

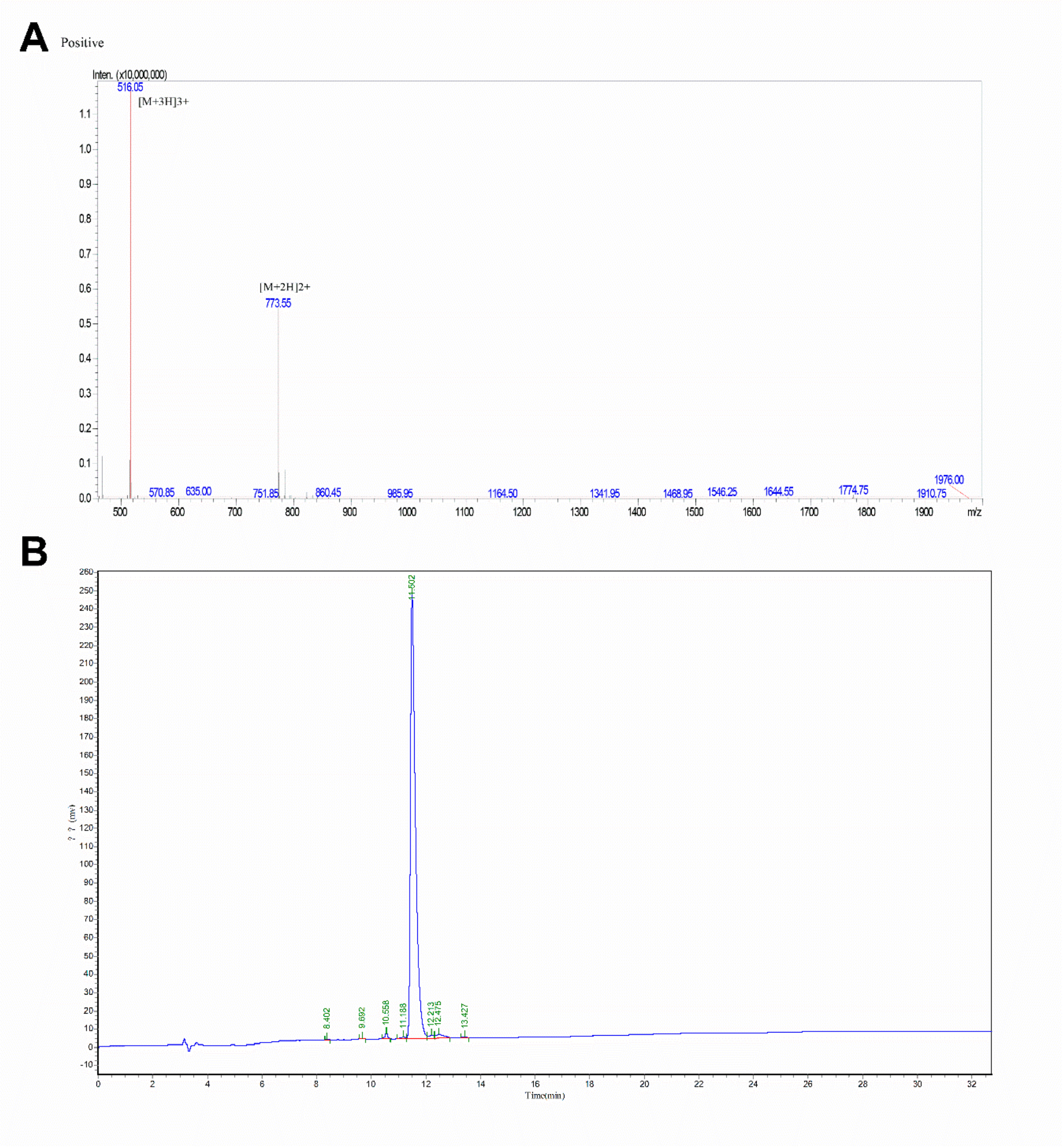

Figure S5. The mass spectrum (A) and RP-HPLC chromatogram (B) of GHaR9R.

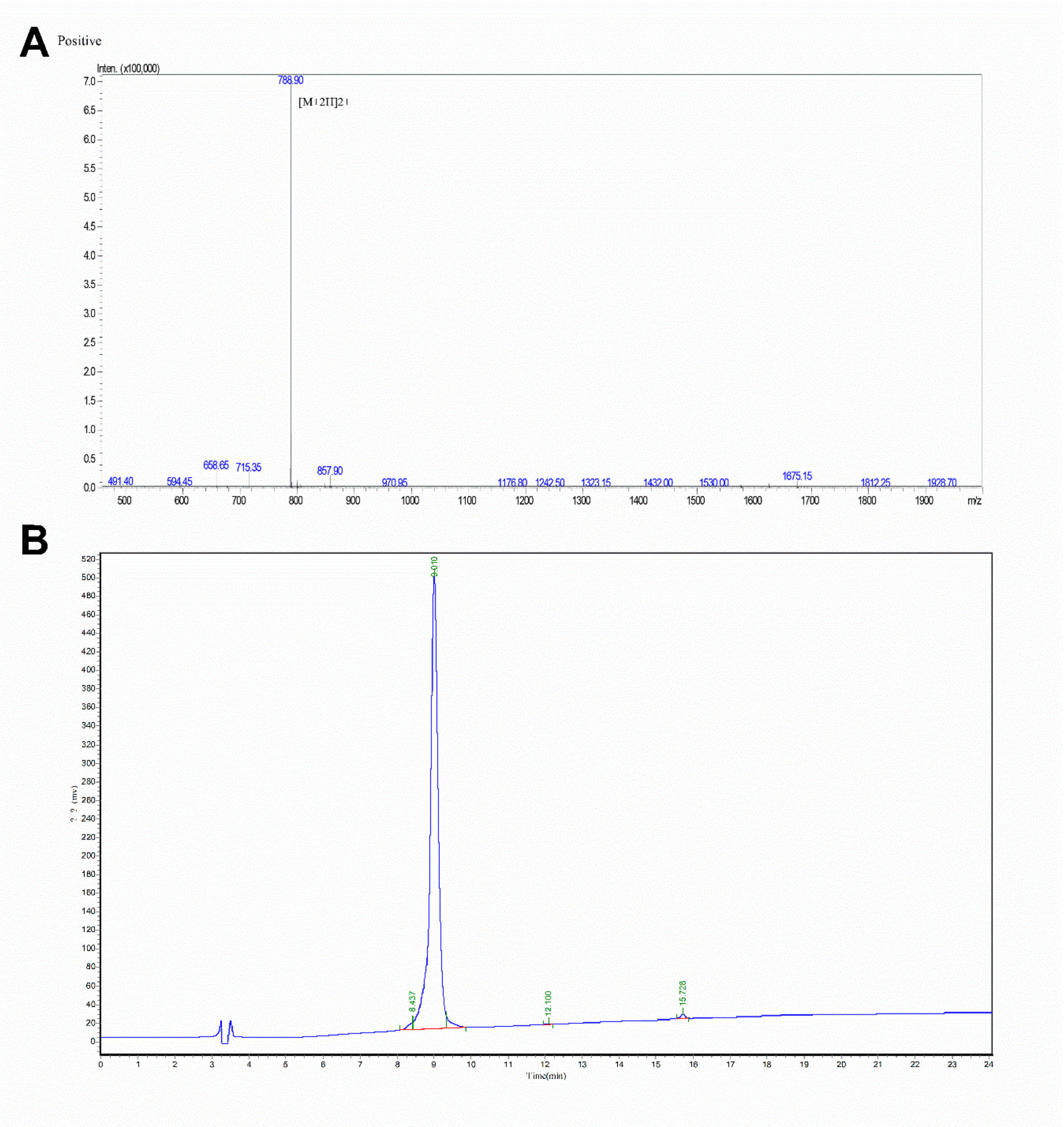

Figure S6. The mass spectrum (A) and RP-HPLC chromatogram (B) of GHaR9W.

© 2020 by the authors. Submitted for possible open access publication under the terms and conditions of the Creative Commons Attribution (CC BY) license (http://creativecommons.org/licenses/by/4.0/).
